# Supplementary material for: Aqueous Two‐Phase Bioinks for Discrete Packing and Compartmentalization of 3D Bioprinted Cells
Source: Adv Healthc Mater. 2026 Feb 18;15(25):e03219. doi: 10.1002/adhm.202503219 (PMC13331587; doi:10.1002/adhm.202503219)
Supplement: Supplementary file 1 — Supporting File: adhm70953‐sup‐0001‐SuppMat.docx. [file ADHM-15-0-s001.docx]

Supporting Information

**Aqueous two-phase bioinks for discrete packing and compartmentalisation of 3D bioprinted cells**

*Martina Marcotulli, Arianna Iacomino, Federico Serpe, Lucia Iafrate, Marco Bastioli, Giorgia Montalbano, Biagio Palmisano, Silvia Franco, Roberta Angelini, Alessandro Corsi, Mara Riminucci, Giancarlo Ruocco, Chiara Scognamiglio, Andrea Barbetta*, and Gianluca Cidonio**


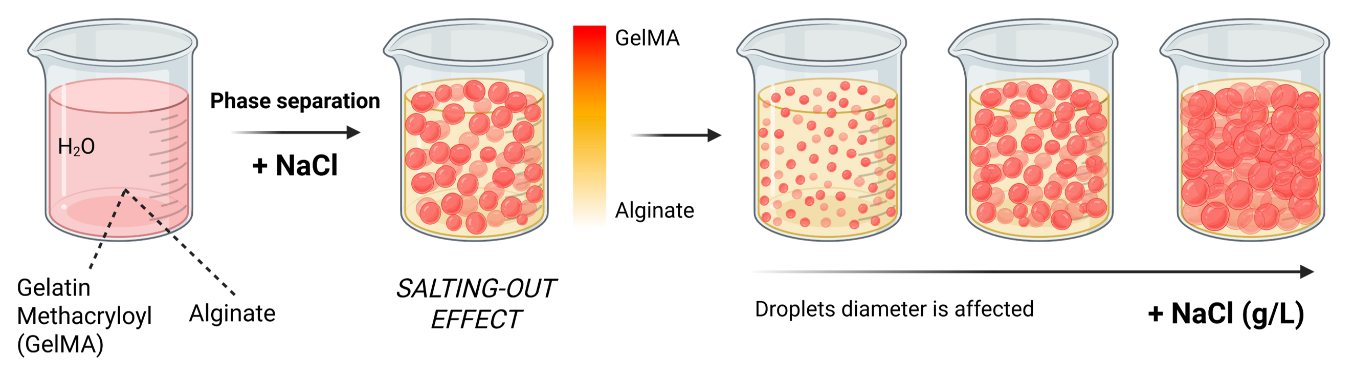


**Figure S1.** Schematic representation of the salting-out effect in the ATPS bioink system. The presence of NaCl in the bioink solution enhances the ionic strength of the medium, which reduces the miscibility between GelMA and alginate phases. The process increases interfacial tension, promoting the formation of GelMA-rich droplets (dispersed phase) within the alginate-rich continuous phase. The schematic illustrates how higher salt concentrations lead to more pronounced phase separation and larger dispersed domains.


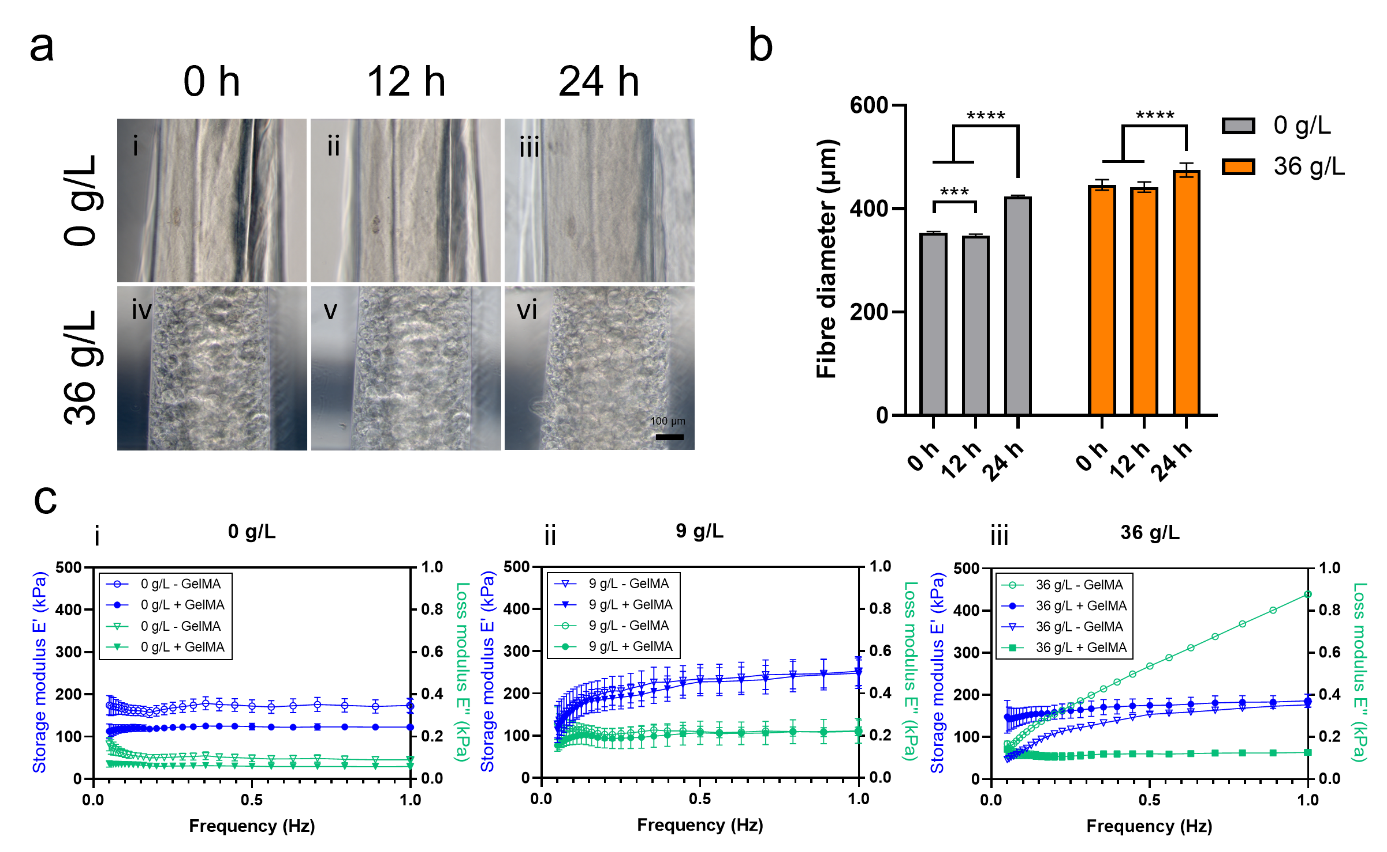


**Figure S2**. Swelling and mechanical analysis of printed ATPS fibres. a) Images of fibres swelling at 0 g/L NaCl taken a-i) 0 hours, a-ii) 12 hours, a-iii) 24 hours and fibres at 36 g/L NaCl taken a-iv) 0 hours, a-v) 12 hours and a-vi) 24 hours. b) Quantification of printed fibres swelling at 0 - 36 g/L NaCl. c) E' and E'' values obtained from compression tests performed on samples at c-i) 0, c-ii) 9 and c-iii) 36 g/L with and without GelMA cross-linking. Scale bar: (a) 100 μm. Statistical significances were assessed by one-way ANOVA. Mean ± S.D. n=3, ****p<0.0001, ***p<0.001.


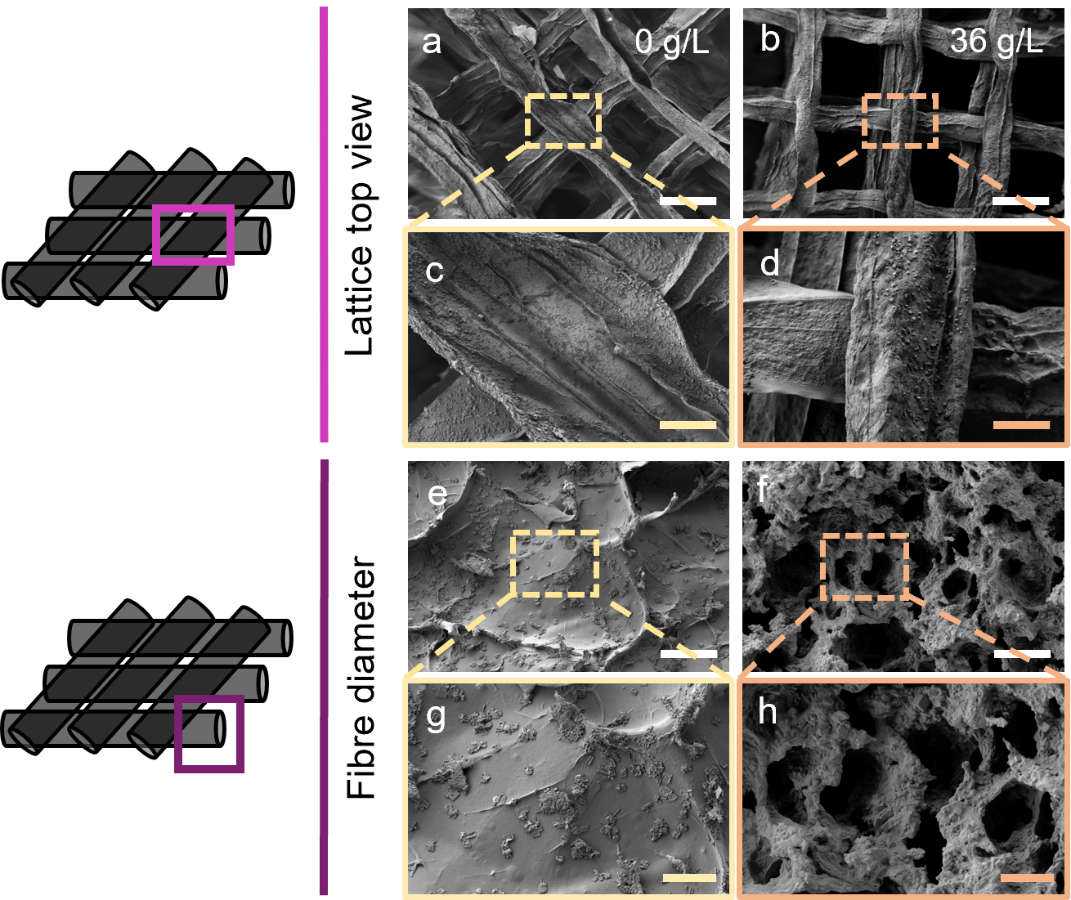


**Figure S3**. SEM characterisation of ATPS scaffolds. SEM top view images of the freeze-dried fibres at i, ii) 0 g/L NaCl and iii-iv) 36 g/L NaCl. SEM images of sections of lyophilised fibres at v-vi) 0 g/L NaCl and vii-viii) 36 g/L NaCl. Scale bars: (a,b) 500 μm, (c,d) 100 μm, (e,f) 100 μm, (g,h) 50 μm. Mean ± S.D. n=3.


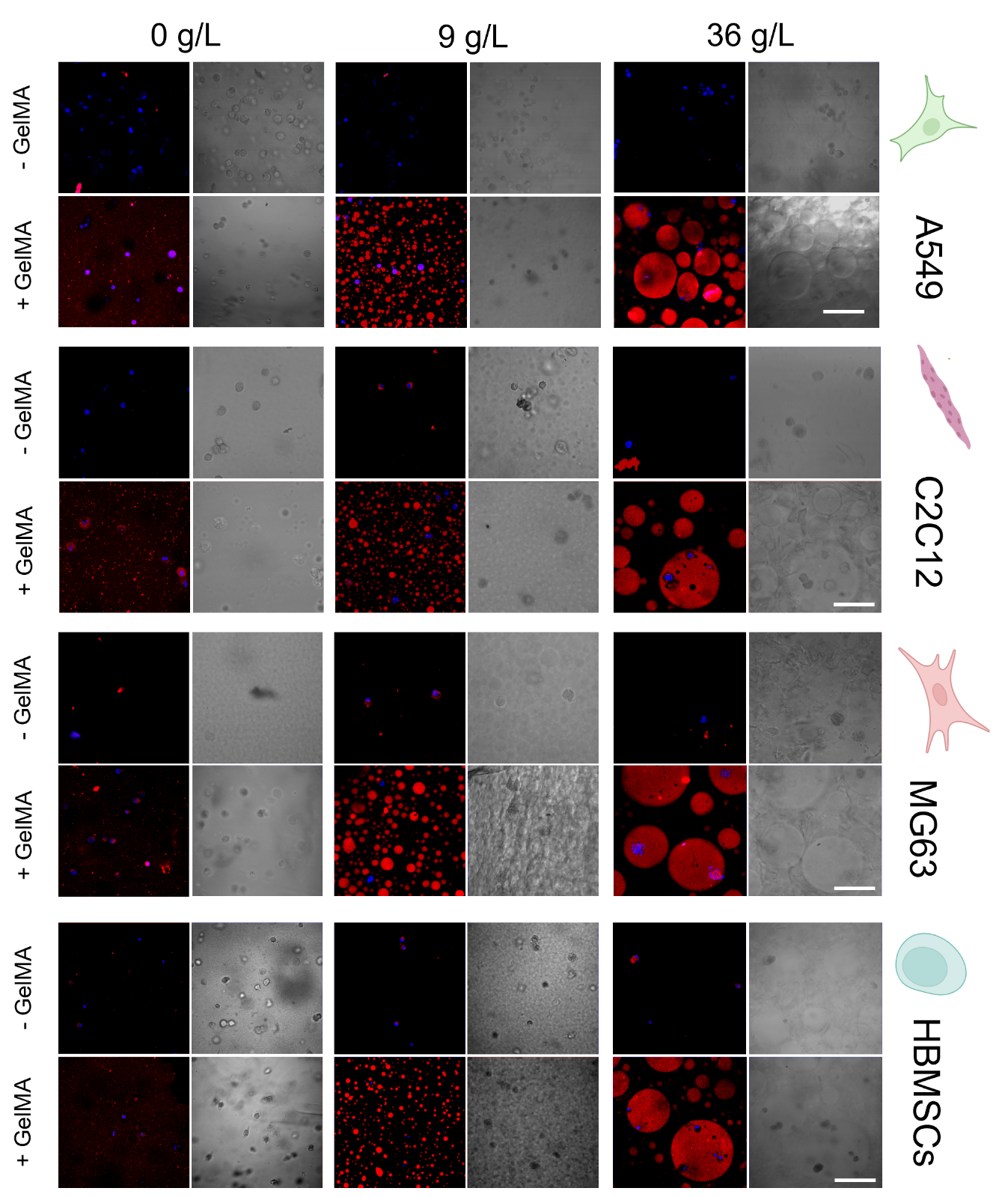


**Figure S4**. Partitioning of different cell types within the various ATPS scaffold formulations. Brightfield and confocal (merged) images representative of cells labelled with DAPI to show the nucleus (blue) of the different cell types (A549, C2C12, MG63, HBMSCs), and GelMA marked with rhodamine B to show the inner phase (red) of the scaffolds at 0 - 9 - 36 g/L. In all cell types, images were taken both under conditions where GelMA was not chemically cross-linked (-GelMA) and under conditions where GelMA was chemically cross-linked (+GelMA). Scale bars: (a, b, c, d) 100 μm. Mean ± S.D. n=3.


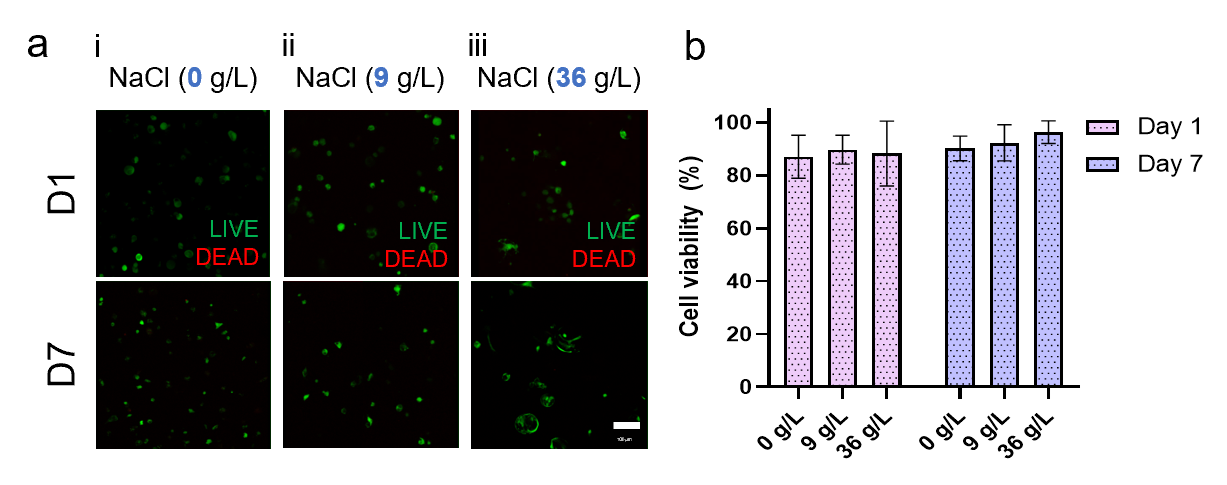


**Figure S5**. Cell viability of HBMSCs encapsulated in ATPS scaffolds. a) Confocal images with HBMSCs cells labelled with Calcein to show live cells (green) and cells labelled with propidium iodide to show dead cells (red) for the conditions a-i) 0 g/L NaCl, a-ii) 9 g/L NaCl and a-iii) 36 g/L NaCl. b) Quantification of cell viability at day 1 and day 7 for samples 0 - 9 - 36 g/L. Scale bar: (a) 100 μm. Statistical significances were assessed by two-way ANOVA. Mean ± S.D. n=3.
